# Supplementary material for: Increased rates of unattained developmental milestones among Israeli children between 2016 and 2020: a national report
Source: Isr J Health Policy Res. 2023 Dec 21;12:38. doi: 10.1186/s13584-023-00586-5 (PMC10740256; doi:10.1186/s13584-023-00586-5)
Supplement: Supplementary file 1 — Additional file 1: Supplemetary figures. [file 13584_2023_586_MOESM1_ESM.docx]

**Supplementary figures**


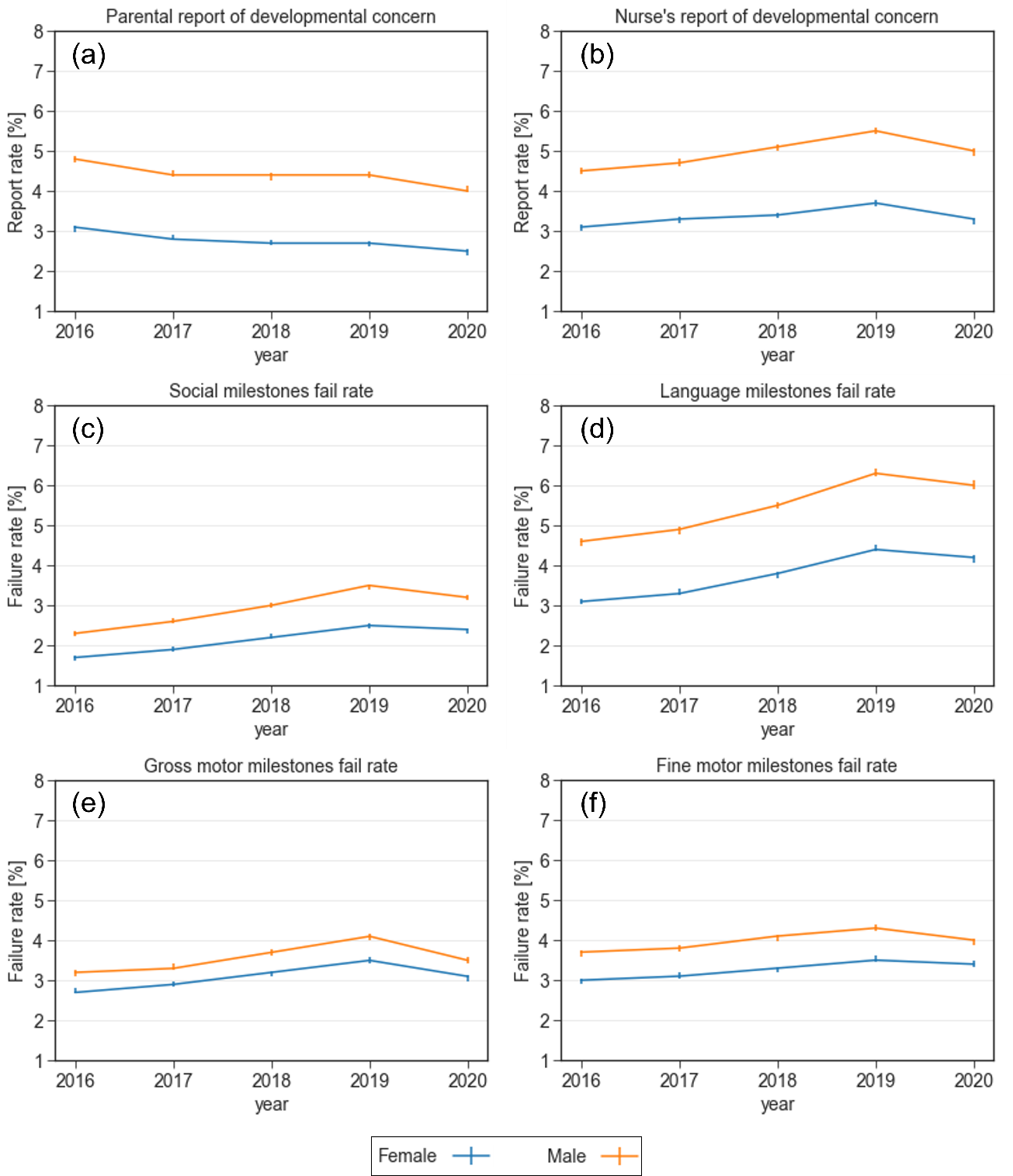


Supplementary Figure 1. Reports of concern and failure rates in various developmental domains stratified by child’s sex between 2016-2020. (a) Parental report of developmental concern (b) Nurse's report of developmental concern (c) Failure rate in the social domain (d) Failure rate in the language domain (e) Failure rate in the gross motor domain (f) Failure rate in the fine motor domain


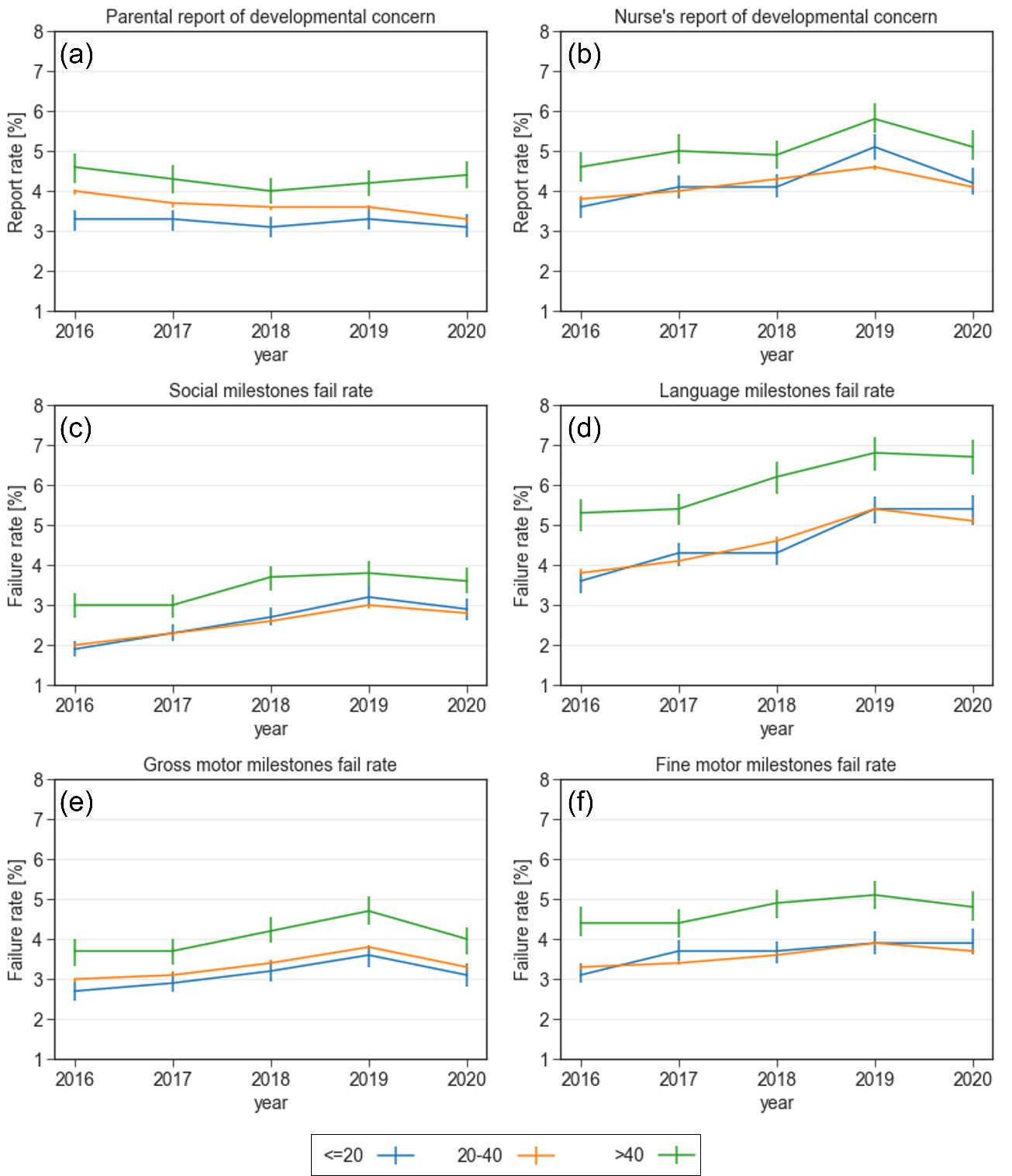


Supplementary Figure 2. Reports of concern and failure rates in various developmental domains stratified by maternal age between 2016-2020. (a) Parental report of developmental concern (b) Nurse's report of developmental concern (c) Failure rate in the social domain (d) Failure rate in the language domain (e) Failure rate in the gross motor domain (f) Failure rate in the fine motor domain


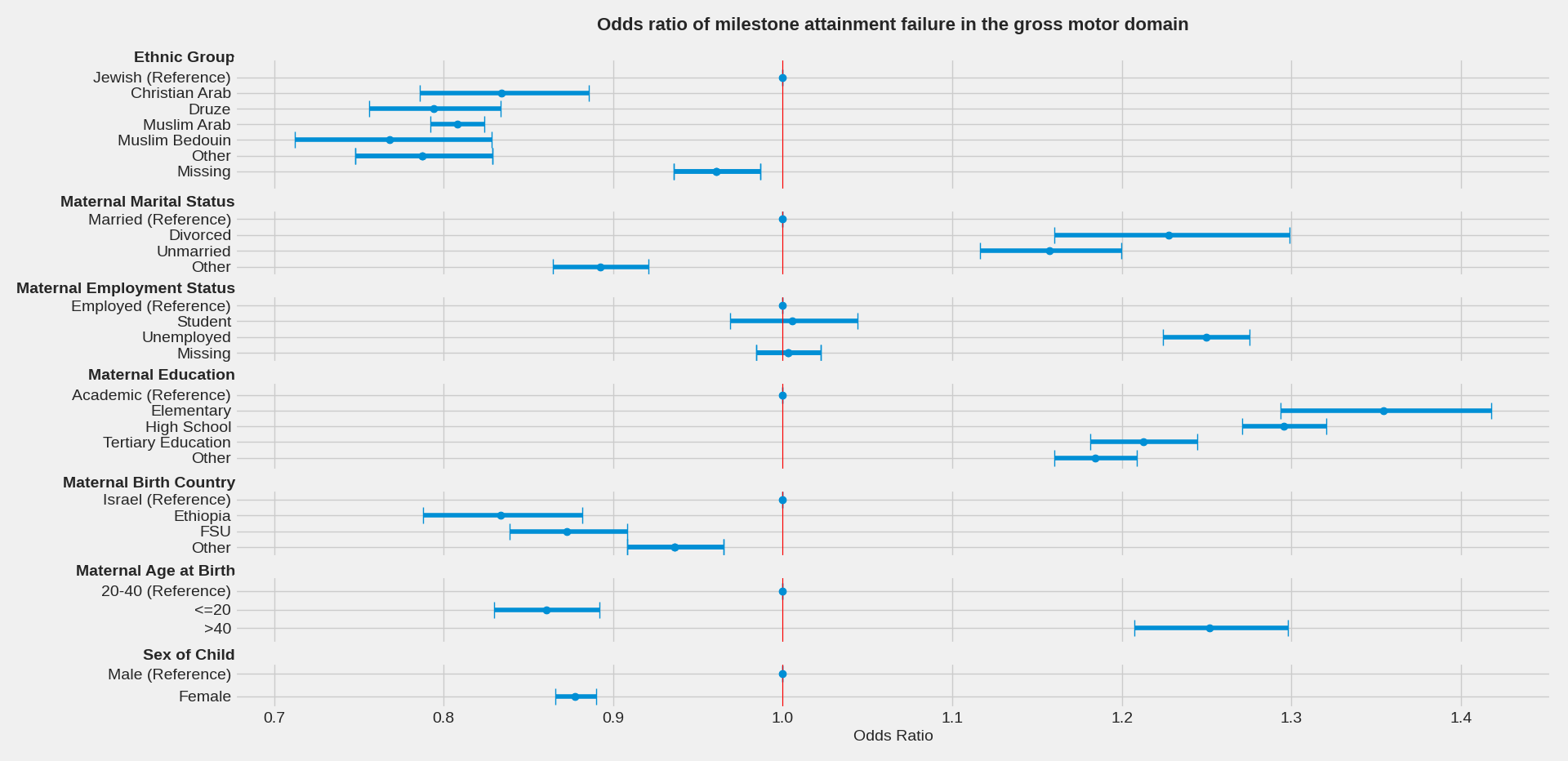


Supplementary Figure 3. Mutually adjusted odds ratios of milestone attainment failure in the gross motor domain, by demographic factors

**
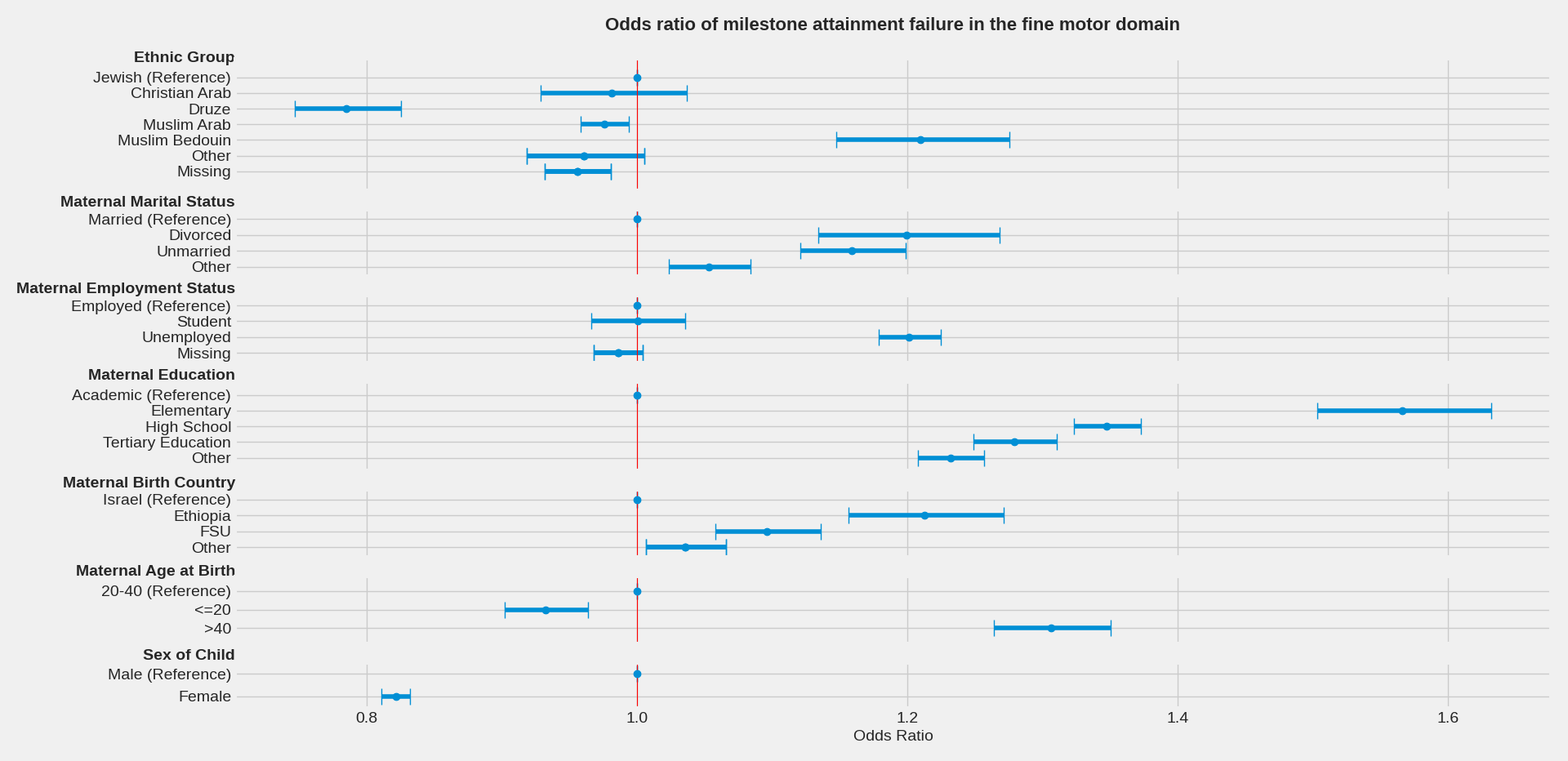
**

Supplementary Figure 4. Mutually adjusted odds ratios of milestone attainment failure in the fine motor domain, by demographic factors


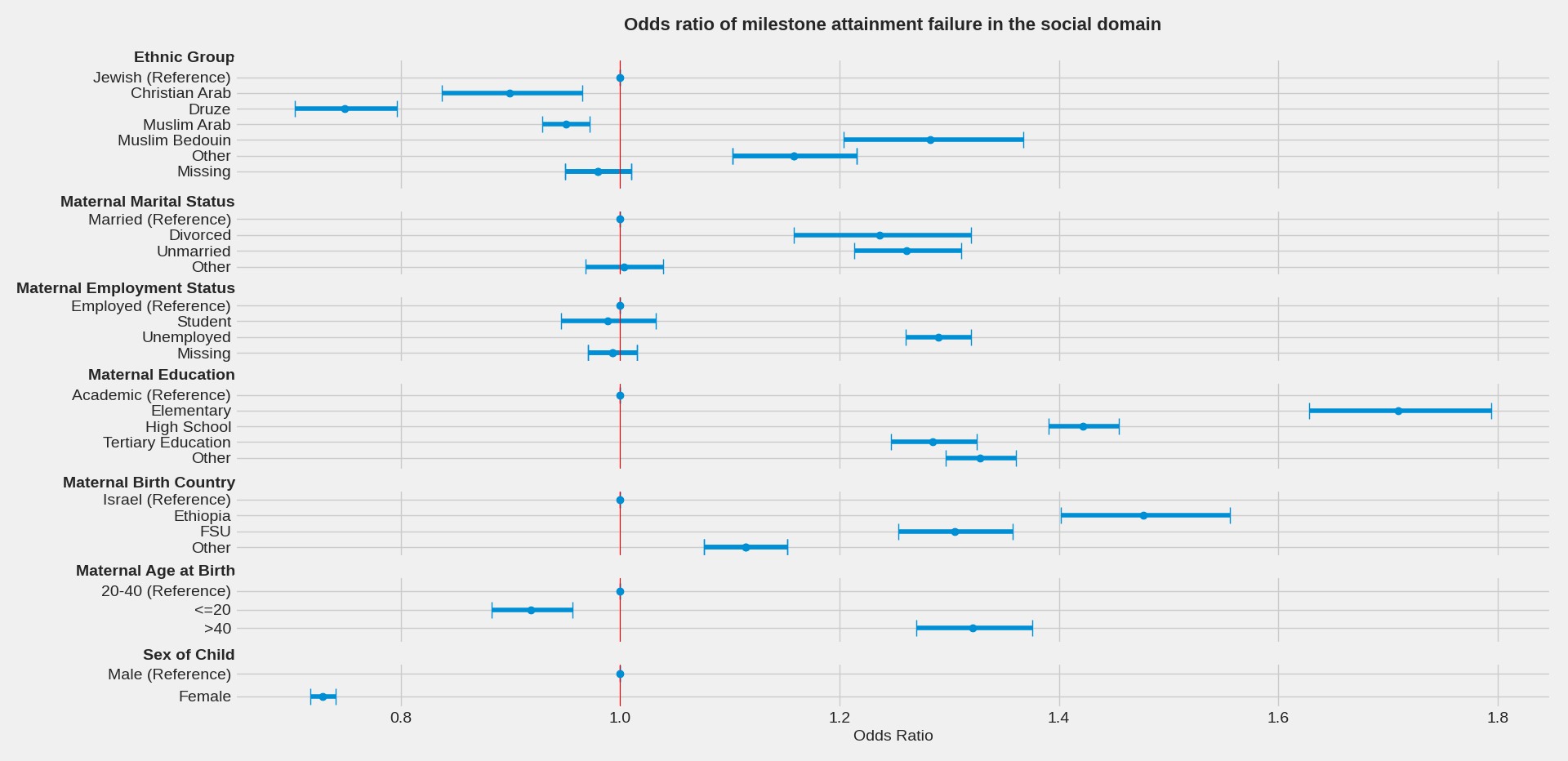


Supplementary Figure 5. Mutually adjusted odds ratios of milestone attainment failure in the social domain, by demographic factors


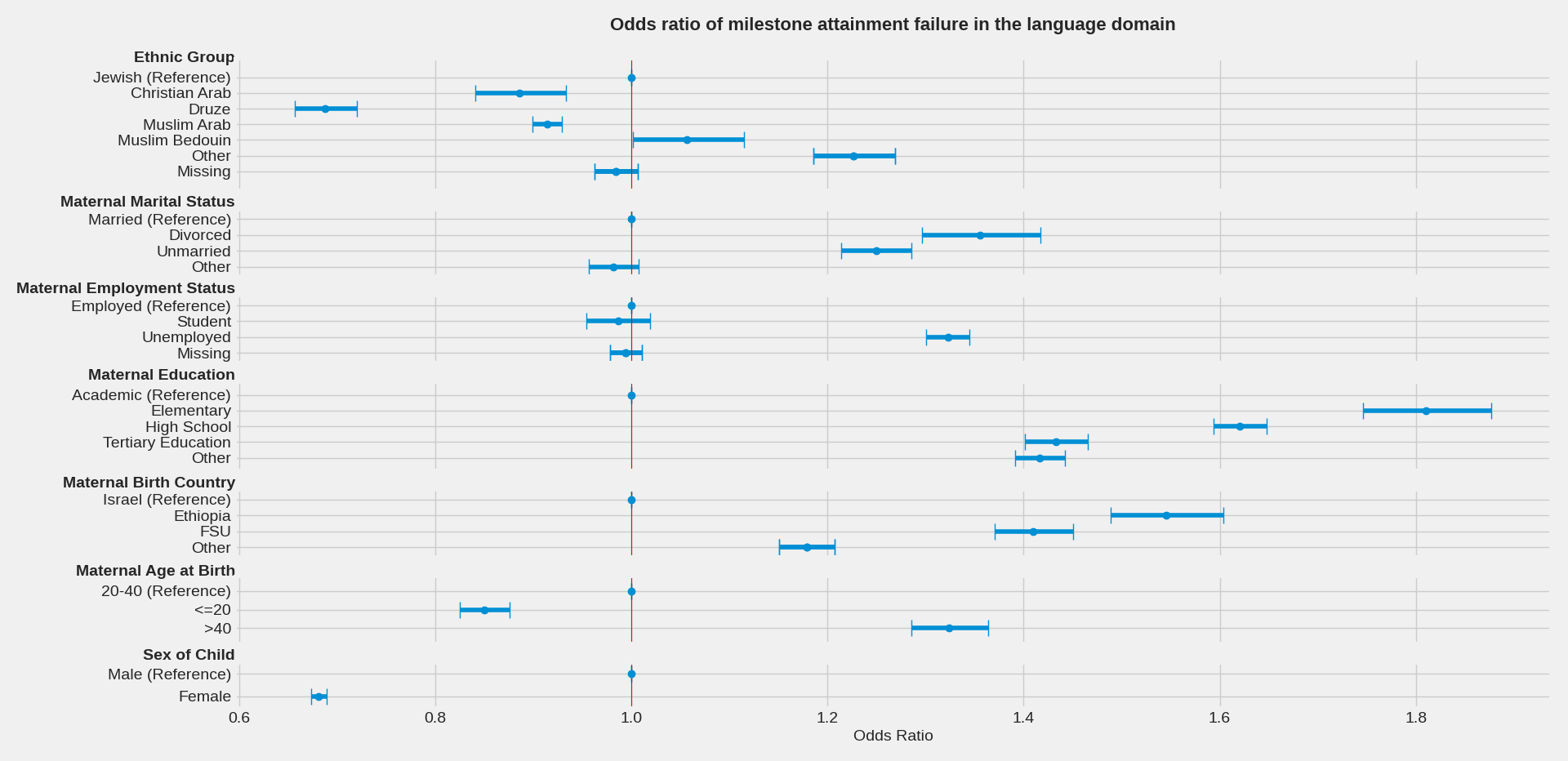


Supplementary Figure 6. Mutually adjusted odds ratios of milestone attainment failure in the language domain, by demographic factors
